# Dental pulp stem cells conditioned medium-functionalized microspheres for endodontic regeneration

**DOI:** 10.3389/fcell.2025.1627220

**Published:** 2025-07-17

**Authors:** Jing Gao, Yuhan Wang, Tongxing Zhang, Jiafei Qu, Qian Liu, Yao Chen, Baoshan Xu, Jing Shen

**Affiliations:** ^1^Department of International VIP Dental Clinic, Tianjin Stomatological Hospital, School of Medicine, Nankai University, Tianjin, China; ^2^ Tianjin Key Laboratory of Oral and Maxillofacial Function Reconstruction, Tianjin, China; ^3^Department of Minimally Invasive Spine Surgery, Tianjin Hospital, Tianjin University, Tianjin, China; ^4^Stomatology Department, Tianjin Ninghe District Qilihai Hospital, Tianjin, China; ^5^Department of Operative Dentistry and Endodontics, Tianjin Stomatological Hospital, School of Medicine, Nankai University, Tianjin, China

**Keywords:** conditioned medium, microspheres, dental pulp stem cells, odontogenic differentiation, endodontic regeneration

## Abstract

**Introduction:**

Dental pulp disease represents a prevalent oral pathology with limited success in functional regeneration of the pulp-dentin complex. Stem cell-based endodontic regenerative therapy has emerged as a promising approach, leveraging the principles of tissue engineering through the combination of stem cells, scaffolds, and growth factors. However, recreating the native pulp microenvironment remains a critical challenge.

**Methods:**

This study developed a novel strategy to mimic the dental pulp regeneration microenvironment using nucleus pulposus microspheres (NPM) loaded with conditioned medium (CM) for dental pulp stem cells (DPSCs). The biocompatibility of NPM and the effects of NPM-loaded CM on DPSCs differentiation and angiogenesis were systematically evaluated. A semi-isotopic in vivo model was employed to assess pulp-like tissue regeneration.

**Results:**

NPM exhibits good biocompatibility, and NPM-loaded CM enhances the odontogenic differentiation and angiogenic potential of DPSCs. Furthermore, the DPSCs+ NPM + CM complexes promoted the regeneration of pulp-like tissue in an in vivo semi-isotopic model. Mechanistically, key bioactive cues secreted in the CM mediated the multi-directional differentiation potential of DPSCs, thereby facilitating pulp tissue regeneration.

**Discussion:**

The 3D pulp-specific microenvironment, facilitated by NPM and CM bioactive factors, enables the regeneration of the pulp-dentin complex, offering a novel strategy and experimental basis for pulp regeneration.

## 1 Introduction

Currently, root canal therapy remains the most effective therapeutic option for periapical disease. However, the tooth after treatment loses its dental pulp, which is critical for tooth nutrition, sensation and vitality, making it susceptible to fracture from the chewing forces (Su et al., 2025). Functional dental pulp regeneration represents a promising therapeutic approach for endodontic disease (Wang et al., 2024). Dental pulp stem cells (DPSCs) possess odontogenic differentiation potential, facilitating dentin and pulp tissue regeneration. Therefore, dental stem cells-mediated pulp regeneration and revascularization have garnered significant attention as potential strategies for revitalizing non-vital teeth (Kwack and Lee, 2022).

Dental pulp stem cells have documented potential in stem cell-based regenerative endodontic therapy as seeding cells (Alothman et al., 2024). After implantation, DPSCs can boost dental pulp regeneration with physiological pulp structure. Growth factors can direct the differentiation of DPSCs, one of the key elements of pulp regeneration (Chi et al., 2022). There are many types of growth factors involved in the regulation of pulp regeneration, which can exert synergistic effects, and the use of a single growth factor may have limited effects (Liang et al., 2021). The conditioned medium (CM) of dental pulp stem cells (DPSCs), which is the supernatant collected from cell culture media, is rich in tooth-derived bioactive factors. It can induce multidirectional differentiation of DPSCs *in vitro*, making it a promising candidate for dental pulp tissue engineering (Zhou et al., 2021).

Serving as a critical component in tissue engineering, scaffolds can offer a 3D architecture to support the regenerative microenvironment and sustain stem cell viability and functionality (Noohi et al., 2022). The narrow environment of the root canal where the pulp is located poses great challenge of developing scaffold materials for pulp regeneration (Han et al., 2023). Injectable hydrogels, showing feasibility in the root canal system, may induce ischemic necrosis after use considering their inadequate blood supply and nutrients in the structurally dense central region (Tang et al., 2020). Meanwhile, prefabricated or rigid scaffolding materials, decellularized dental pulp, for example, is impossible to implant into narrow and curved root canals owing to fixed shape (Khurshid et al., 2022). Microspheres are a new type of scaffold for stem cell culture and delivery, which are suitable for complex root canal systems considering their small size, large specific surface area, minimally invasive injectability and many other advantages. Microspheres can provide a 3D microenvironment similar to a natural extracellular matrix (ECM), in addition to the fact that microsphere systems have enhanced cell viability, outperforming conventional block hydrogels (Zheng et al., 2023). With a long-term application in regenerative medicine, ECM scaffolds have been documented to offer excellent injectability and adjustable mechanical properties, becoming a perfect material option for endodontic regenerative scaffolds (Bakhtiar et al., 2022). Various successful cases have been reported concerning the use of decellularized scaffolds in human (Yang et al., 2024), porcine (Fu et al., 2020), or bovine pulp (Bakhtiar et al., 2020) tissues in promoting pulp regeneration. So far, there is limited availability of allogeneic or xenogeneic pulp tissues. The nucleus pulposus within the intervertebral disc is rich in proteoglycan and type II collagen, and has an abundance of highly hydrophilic ECM components. The nucleus pulposus decellularized ECM hydrogel has been discovered to promote tissue-specific differentiation and tissue regeneration of bone marrow mesenchymal stem cells (Peng et al., 2021). Therefore, this study collected bovine-derived medullary tissue for further experiments, considering that it can be harvested in large quantities from animals at a relatively low production cost, without ethical constraints. In this study, the decellularized ECM scaffolds of bovine medullary nucleus tissue were prepared into microspheres as an alternative scaffold for dental pulp regeneration.

**FIGURE 1:**
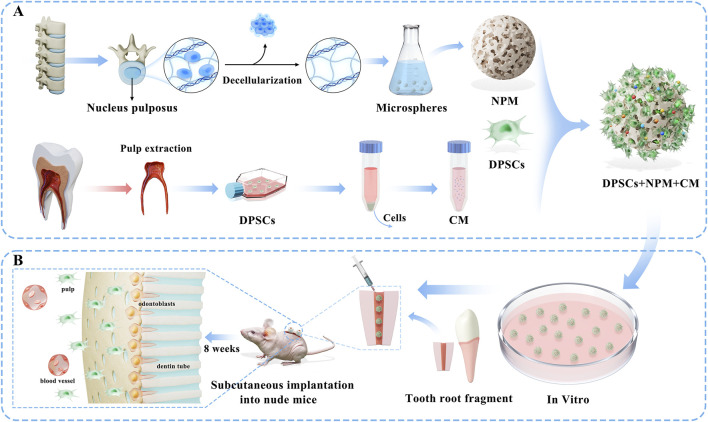
Schematic diagram of the project flow scheme: **(A)** First, decellularized extracellular matrix scaffolds from bovine nucleus pulposus tissues were fabricated into microspheres (NPM) to serve as regenerative scaffolds, while conditioned medium (CM) containing multiple bioactive factors was collected from dental pulp stem cells (DPSCs), followed by the construction of DPSCs+NPM+CM complexes. **(B)** Systematic in vitro evaluations were conducted to assess the biocompatibility of NPM and the ability of DPSCs to proliferate, odontogenic differentiate, and vascularize toward differentiation under different concentrations of CM factors. *In vivo* assessment of pulp regeneration efficacy through implantation of DPSCs+NPM+CM complexes in an immunodeficient nude mouse semi-orthotopic model.

By integrating nucleus pulposus microspheres (NPM) with CM, injectable pulp regeneration scaffolds were scheduled to be constructed to adapt to complex root canal systems. Our emphases were the biocompatibility of NPM, as well as the ability of DPSCs to proliferate and differentiate odontogenetically under different concentrations of CM factors. The effect of pulp regeneration *in vivo* was also evaluated by implanting DPSCs + NPM + CM complexes in a mouse model (Figure 1).

## 2 Materials and methods

### 2.1 Fabrication and characterizations of NPM

The nucleus pulposus ECM was obtained from fresh bovine-derived intervertebral disc nucleus pulposus tissues by programmed decellularization technique as previously reported (Ma et al., 2024). After decellularization of the nucleus pulposus tissues, we used H&E staining, DNA quantification and collagen quantification methods to identify the extent of decellularization of the medullary tissue to ensure that all cellular components, including DNA and other substances, were removed while maximizing the retention of ECM components. By employing modified electrostatic printing combined with freeze-drying technique, NPM were prepared as follows: firstly, the nucleus pulposus ECM was dissolved into ECM solution by using acid solubilization and enzyme digestion method, ECM solutions of 0.5%, 1%, and 1.5% were prepared according to mass concentration, with the Ph value adjusted to be neutral; then, the 0.5%, 1%, and 1.5% ECM solution was transferred to a syringe with 24G metal syringe needle. Then the syringe was fixed on the micro-propulsion pump, the distance of the needle from the liquid nitrogen receiving bath was 15 cm, and the extrusion rate of the solution was set to 10 mL/h; the positive pole of the high-voltage power supply was connected to the needle of the syringe, and the negative pole was connected to the container containing liquid nitrogen, and the voltage was set to 10 KV. The ECM droplets were collected by using a liquid nitrogen bath, and the freeze-cured NPM were freeze-dried to obtain the primary NPM; finally, NPM preparation was completed after secondary freeze-drying through the EDC + NHS cross-linking denaturation. The parameter settings of the freeze-drying machine used in this study are as follows: temperature set to −50°C, pressure set to less than 10 Pa, and freeze-drying time set to 48 h.

The overall morphology and surface microstructure of NPM were observed under in-body microscope and scanning electron microscope (SEM). The size and pore size of NPM prepared with different parameters were measured to screen out the optimal preparation NPM condition. The degradation curves of NPM were plotted after the measurement of the degradation properties of NPM.

### 2.2 Culture of DPSCs

DPSCs were sourced from premolar teeth extracted from 18 to 22-year-old fully-informed patients for orthodontic reasons in the Maxillofacial Surgery Clinic of Tianjin Stomatological Hospital. Isolated teeth were then placed in sterile extraction solution, and rinsed repeatedly with 10% penicillin-streptomycin-added phosphate buffer. Pulp tissues were removed, cut into small pieces and transferred to a new sterile medium, evenly adhered to the culture flask bottom. Tissues were cultured supplemented with α-MEM medium containing 20% fetal bovine serum (FBS, Gibco), and 1% penicillin-streptomycin (PS, Beyotime) at 37°C in a 5% CO_2_ incubator (Raik et al., 2023).

### 2.3 Preparation of CM

DPSCs from at passages 3–5 (P3–5) were selected based on the optimal viability, transferred into T75 culture flasks, and incubated at 37°C under 21% O_2_ for 24 h to allow facilitate adherent growth. When the cell confluence was 70%, the culture was continued by replacing the fresh serum-free α-MEM medium containing only 1% PS without FBS (Chen et al., 2024). The day the serum-free medium was changed was recorded as day 0. The supernatants of the CM-containing medium produced by DPSCs were collected at 1, 3, and 5 days, respectively. The collected CM was centrifuged and filtered to remove cellular debris and contaminants, and the harvested samples were placed at −80°C for subsequent experiments within 1 month.

### 2.4 Characterizations of cell adhesion of DPSCs on NPM

The pre-treated NPMs were first immersed in ɑ-MEM medium containing 10% FBS and 1% PS and then transferred to confocal dishes (glass-bottom culture dishes, America) for 10 min to allow the microspheres to settle naturally at the bottom of the dish. To avoid the separation of microspheres caused by mechanical disturbance, after absorbing the upper medium, we gently added the DPSC suspension into the dish to ensure that the cells effectively contacted the deposited NPM surface, and cultured under 37°C and 21% O_2_ conditions for 48 h. After fixation with 4% paraformaldehyde, cells were permeabilized with 0.5% Triton X-100 for permeabilization. The cytoskeleton was stained using FITC using FITC to stain the cytoskeleton and the nuclei were stained with DAPI sealer under light-avoidance conditions. Finally, the adhesion of DPSCs on NPM was observed under a confocal fluorescence microscope.

### 2.5 *In vitro* osteogenic and odontogenic differentiation of DPSCs + NPM with CM

Assessment of the proliferation of DPSCs quantitatively was performed using CCK-8 (KeyGen, China). DPSCs in the logarithmic phase of P3-5 were inoculated in 96-well plates covered with NPM at 5,000 cells/well, divided into blank, 1 day CM, 3 days CM, and 5 days CM groups. 100 μL of the mix (comprising 50 µL of complete medium and 50 µL of CM-conditioned medium) was added to each well, and the medium was changed every 3 days. The day of the first addition of the conditioned medium was designated as day 0, and cell proliferation was dynamically monitored using the CCK-8 assay on subsequent days (1, 3, 5, and 7). After 1 day, 3 days, and 5 days of culture, cells were added with CCK-8 solution for 1.5 h of incubation at 37°C. The cell viability was determined by the absorbance measured at 450 nm using enzyme marker.

To assess the promotional activity of CM on the odontogenic differentiation of DPSCs, quantitative reverse transcription PCR (RT-qPCR) was adopted to measure the mRNA expression levels of DSSP and DMP-1. DPSCs were stimulated with α-MEM medium as the control group, and 1 day CM, 3 days CM, and 5 days CM as the experimental group, which were induced to culture for 7 days and 14 days, respectively. DPSCs cultured under different conditions were collected at different time points, followed by the extraction of the total RNA of each sample using an RNA extraction kit (Agbio, Hunan). Then, a reverse transcription kit (Agbio, Hunan) to attain cDNA, and the resulting cDNA was collected for subsequent detection.

DPSCs in good condition of P3-5 were selected and transferred to 12-well plates covered with NPM. α-MEM medium was used as the blank control group, osteogenic induction solution as the positive control group, and CM collected on days 1, 3, and 5 as an experimental group for stimulation of DPSCs. Osteogenic induction components were added to the 1 day CM group, 3 days CM group, and 5 days CM group medium groups. After 7 days of induction, alkaline phosphatase (ALP) expression was shown by staining using an ALP chromogenic kit, after which ALP activity was analyzed quantitatively. After 14 days of induction, staining was performed using alizarin red to observe mineralized nodule formation microscopically. With the dissolution of samples with 95% ethanol, their optical density was measured at 562 nm quantitatively.

### 2.6 *In vitro* angiogenic potential of CM

To assess the angiogenic activity of CM, an *in vitro* tubule formation assay was performed on HUVECs. Matrigel (ABW-Bio, Shanghai, China) was used to uniformly cover the bottom of a 48-well plate, and incubated at 37°C for 30 min to solidify. HUVECs were digested, resuspended, and then inoculated into the well plates. The control group was established by stimulating HUVECs with serum-free α-MEM medium, whereas the experimental group was stimulated by the addition of 1 day, 3 days and 5 days CM. After 0, 3, 6, and 9 h of incubation, the results were observed, photographed, quantitatively analyzed, and counted under an ordinary light microscope (Dawson et al., 2011). VEGF genes expression was detected via RT-qPCR.

### 2.7 *In vivo* pulp-like tissue regeneration in immunodeficient nude mice model

Root segments were prepared by collecting mature single-rooted premolar teeth from adult patients without any root canal intervention. Root segments approximately 5–6 mm in length were intercepted from the middle of the root, with the removal of pulp tissue and part of the dentin. Then, the root canals were enlarged to 3 mm in diameter and ultrasonicated for 10 min after 5 min of treatment using 17% EDTA. Subsequently, the roots were disinfected through 10 min of processing with 5.25% sodium hypochlorite. Finally, the processed roots were incubated for 3–7 days at 37°C, after being rinsed with a sterile PBS solution, to ensure that the disinfectant was completely removed (Atila et al., 2022).

Following approval by the Ethics Review Committee of Tianjin Stomatological Hospital, male BALB/c nude mice (20–25 g, 5–6 weeks old) were randomly divided into experimental groups with 3 mice per group for the *in vivo* study. Seven groups were created with different treatment protocols. A subcutaneous pouch-like structure was created on the back of nude mice by blunt dissection method. After the implantation of dental root segments into mouse backs, the next step was the injection of 100 μL of Matrigel hydrogel containing the following components into the root segments, i.e., PBS, CM, NPM, DPSCs, DPSCs+5dCM, DPSCs + NPM, and DPSCs + NPM+5dCM groups. Two root segments were implanted in the dorsal skin of each nude mouse, after which the incision was sutured. Eight weeks later, the nude mice were euthanized for harvesting root segments of each group. After immediate fixation in 4% formaldehyde for 24 h, these specimens were decalcified in 10% EDTA for 2 months, embedded in wax blocks, further stained with hematoxylin-eosin (HE) and Masson’s trichrome staining for result visualization under light microscopy (Yang et al., 2021).

### 2.8 Statistical analysis

All data were expressed as mean ± standard deviation (n ≥ 3). The statistical significance, determined by a threshold of *P* < 0.05, was calculated by one-way analysis of variance and Tukey’s test.

## 3 Results

### 3.1 Characterization of NP-ECM and NPM

Compared with natural nucleus pulposus tissue, decellularized nucleus pulposus tissue became loose and transparent, and the tissue hardness decreased (Supplementary Figure S1A,B); The H&E staining results showed that more nuclei were visible in natural nucleus pulposus tissue, while no nuclei were found in decellularized nucleus pulposus tissue (Supplementary Figure S1C,D). This proves that our decellularization process is relatively mature and can thoroughly remove cellular components, including nuclei. The DNA quantification results showed that the DNA content in decellularized nucleus pulposus tissue was significantly lower than that in natural nucleus pulposus tissue, and the difference was statistically significant (*P* < 0.001) (Supplementary Figure S1E). The collagen quantification results showed that the relative content of collagen in decellularized nucleus pulposus tissue was higher than that in natural nucleus pulposus tissue, and the difference was statistically significant (*P* < 0.01) (Supplementary Figure S1F), indicating that the decellularization process can still retain the main components in ECM, which is very important for the preparation of NPM in the later stage.

**FIGURE 2:**
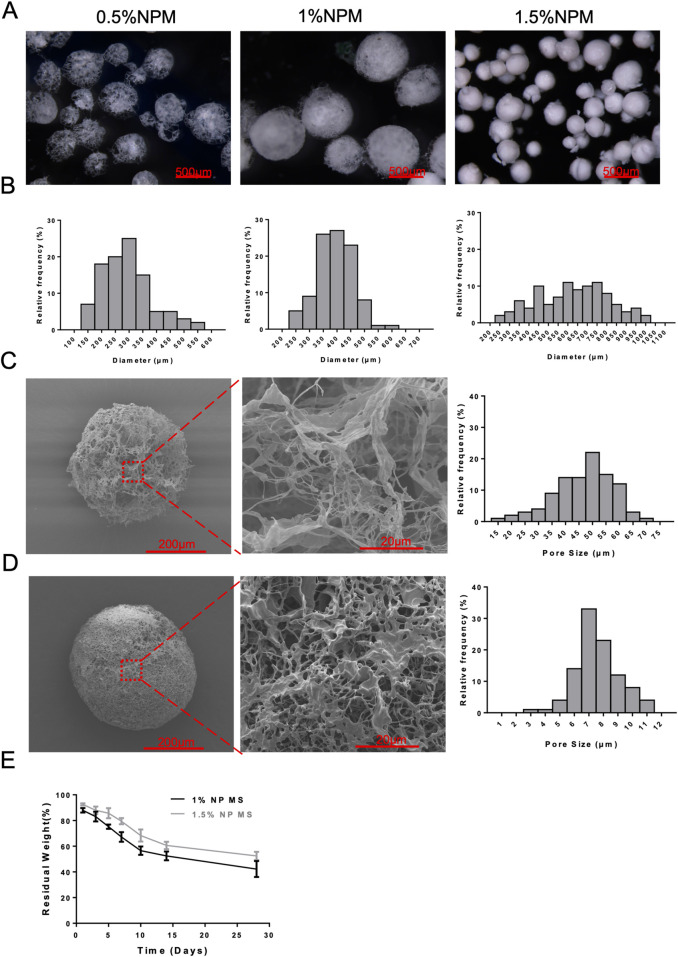
Structural characterization of NPM. **(A)** Light microscopy images of 0.5%, 1%, and 1.5% NPM (scale bar: 500 µm). **(B)** The size distribution results of 0.5%, 1%, and 1.5% NPM. **(C)** SEM images of 1% NPM and results of pore size measurements (scale bar: 200 µm, 20 µm). **(D)** SEM images of 1.5% NPM and results of pore size measurements (scale bar: 200 µm, 20 µm). **(E)** Degradation curves of 1% and 1.5% NPM.

Microscopically, 0.5% of NPM showed a loose and large pore structure, with more pores and larger pore diameters in the microspheres leading to structural instability, and more microspheres collapsed, presenting as an irregular shape of the microspheres. Its microsphere diameters ranged between 200–350 μm statistically, with an average diameter of 292.91 ± 93.39 μm. 1% NPM had a regular spherical structure and relatively uniform microsphere size, and its microsphere diameter ranged between 350–450 μm, with an average diameter of 393.41 ± 66.90 μm. 1.5% NPM showed a relatively dense structure, and irregular shape of microspheres. Microspheres showed fragmentation, while the microspheres prepared under this condition had relatively greater differences in size and dispersed microsphere diameters, and microsphere diameters were distributed in the range of 250–1,000 μm, with an average diameter of 626.69 ± 184.18 μm (Figures 2A,B). SEM images were failed to collected due to the unstable structure of 0.5% NPM, only SEM images of 1% NPM and 1.5% NPM were collected for microstructural analysis. The results revealed that 1% NPM had a relatively regular spherical shape, and the microspheres were of a fibrous and porous structure under high magnification, with relatively large pore sizes. The pore size ranged mainly between 35–60 μm, with the average pore size of 46.49 ± 10.93 μm, which was suitable for cell adhesion and internal infiltration, as well as for use as a cell delivery vehicle. Meanwhile, 1.5% NPM showed a relatively dense structure. Under high magnification, despite the presence of a fibrous porous structure, the pore structure of the microspheres was mainly composed of a large number of small pores, with relatively poorer connectivity between the pores, and the pores were not separated from the pore structure, with relatively smaller pore diameter. The results of pore size measurements showed that the pore sizes were mainly concentrated in the range of 6–10 μm, with an average pore size of 7.57 ± 1.46 μm (Figures 2C,D). Repair materials suitable for tissue regeneration need to have appropriate biodegradability, and those with too fast or too slow biomaterials degradation rates are not conducive to tissue regeneration. Therefore, this study examined the degradation performance of the NPM. Consequently, 1% NPM had a faster degradation rate than 1.5% NPM, possibly attributed to the lax and macroporous structure of 1% NPM that might be more conducive to the degradation of the material. However, 1% NPM could still retain the pore size of the material after 28 days of *in vitro* degradation. In addition, *in vitro* degradation for 28 days still retained close to 50% of the material residue, demonstrating that this degradation process was relatively slow and could provide sufficient time for tissue remodeling, which was advantageous for *in situ* tissue regeneration (Figure 2E).

### 3.2 Biological assessment of NPM

**FIGURE 3:**
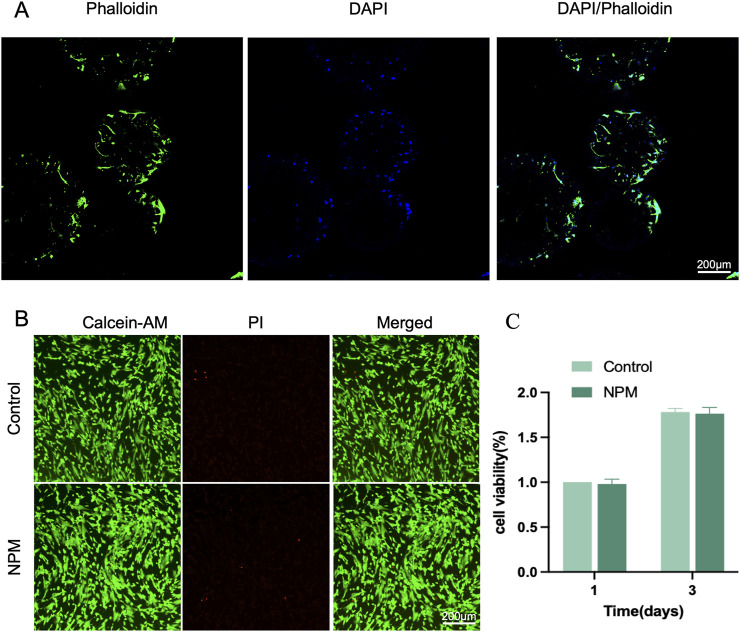
Biological assessment of NPM. **(A)** CLSM images of DPSCs loaded on NPM, cytoskeleton stained with Phalloidin-FITC(green), nuclei stained with DAPI (blue) (scale bar: 200 µm). **(B)** Live-dead staining images (scale bar: 200 µm). **(C)** cell viability analysis.

According to cytoskeleton staining, the attachment of DPSCs to the outer surface of NPM was observed 48 h after cell inoculation. DPSCs were mostly flat-shaped, mostly laying flat on the surface of the microspheres, showing good extension (Figure 3A). There was a close interaction between DPSCs and NPM, and NPM could serve as a good carrier for DPSCs. Meanwhile, the cell proliferation was examined after the inoculation of DPSCs onto the NPM. DPSCs grew rapidly 3 days after cell inoculation (Figure 3C), without significant difference in cell viability compared to normal-grown cells, indicating no significant impact of NPM as carriers on the proliferation of DPSCs. Similarly, under laser confocal microscopy, DPSCs added with NPM extract as culture medium showed strong green fluorescence, and had similar fluorescence intensity with the normal-grown DPSCs group (Figure 3B). Thus, NPM did not negatively affect DPSCs and had good cytocompatibility.

### 3.3 *In vitro* odontogenic differentiation of DPSCs

**FIGURE 4:**
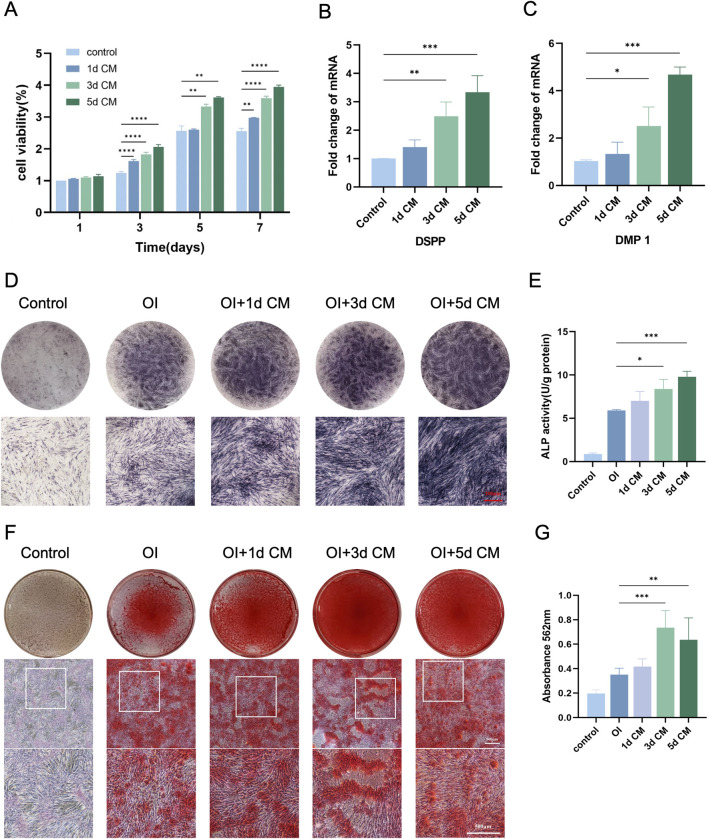
*In vitro* odontogenic differentiation of DPSCs. **(A)** CCK-8 assay for cell proliferation of DPSCs cultured at different time CM. **(B)** DSPP and **(C)** DMP-1 genes expression detected by RT-qPCR. **(D,E)** Representative images of ALP staining and ALP activity of DPSCs after the action of NPM + CM. (Scale bar: 200 µm) **(F,G)** ARS staining and semi-quantitative analysis of DPSCs after the action of NPM + CM. (Scale bar: 500 µm). (*P < 0.05; **P < 0.01; ***P < 0.001; ****P < 0.0001).

In terms of the effect of CM on DPSCs proliferation (Figure 4A), from day 1 to day 7, DPSCs proliferated well in the three groups. The addition of CM significantly accelerated the proliferation of DPSCs during the 7-day incubation. Starting from day 3, the number of cells in CM group was significantly more than that in the control group and peaked at day 7. During culture, 5 days CM again promoted the proliferation of DPSCs, which was better than 3 days CM and 1 day CM, with statistically varied cell volume of cells in the three CMs.

In order to further analyze the ability of dentinogenic differentiation of DPSCs at the genetic level under different culture conditions, DSPP and DMP-1 gene expressions were quantified to investigate the effect of CM on the differentiation of DPSCs into dentinogenic cells (Figures 4B,C). The mRNA expressions of DSSP and DMP-1 were up-regulated obviously in each group of 1 day CM, 3 days CM, and 5 days CM at 7 and 14 days of mineralization induction. Moreover, the mRNA expression level of the 5 days CM group was significantly higher than that of the other two groups (*P* < 0.05).

The initiation of mineralization can be determined by observing the production of ALP, which is essential for the dentin-pulp complex repair process and the formation of tertiary dentin (Wang et al., 2022). Meanwhile, intracellular calcium deposition matrix is also a stage-specific marker for the differentiation of DPSCs into dentinogenic cells. They are abundantly expressed in the early and late stages of dentinogenic cell differentiation, respectively. Thus, this study considered ALP as an early indicator, while calcium deposition as a later indicator of dentinogenic cell activity. Here, dentinogenic dentin cell differentiation was observed by examining ALP activity in DPSCs induced under osteogenic conditions for 7 days and 14 days. After 7 days of CM treatment, ALP activity in DPSCs increased remarkably with increasing time of CM collection, especially under 5 days CM treatment (Figures 4D,E). It could be speculated that CM might contain odontogenic-related bioactive factors that could promote the odontogenic differentiation of DPSCs, and that prolonging the CM collection time was more helpful in promoting the mineralization of DPSCs. After 10 days of osteogenic induction culture, calcium nodules were started to be produced in 1 day CM, 3 days CM, and 5 days CM groups, showing scattered distribution among the cells. After 14 days of osteogenic induction, an overall increase was found in the aspects of red calcium nodule number and diameter in all groups. In relative to the control, the addition of CM for 14 days of induction significantly enhanced the level of mineralized deposition in the cells of the CM group. CM collected for 3 days induced the formation of more calcium nodules compared to those collected for 1 and 5 days (*P* < 0.05) (Figures 4F,G). The observed trend of absorbance change was consistent with the ALP staining results. It could be explained that tooth-derived related bioactive factors in CM can trigger the induction of more DPSCs to differentiate into dentinogenic cells and produce mineralization.

### 3.4 *In vitro* angiogenic potential of CM

**FIGURE 5:**
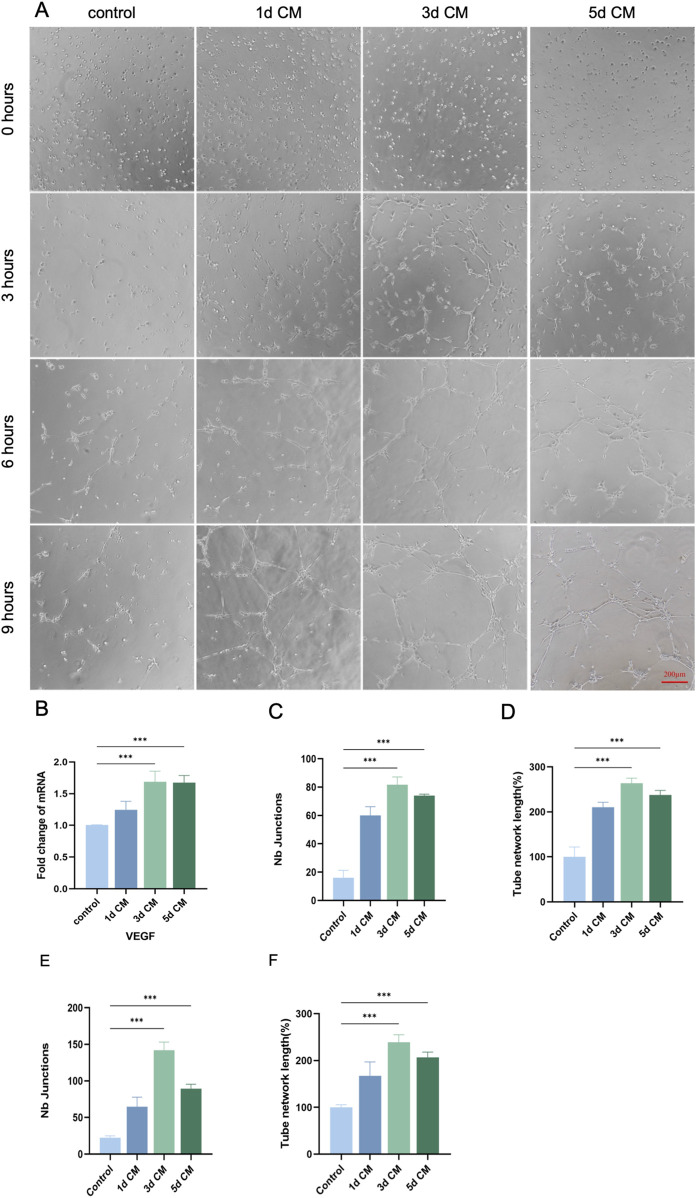
*In vitro* angiogenic potential of DPSCs. **(A)** Representative images of angiogenesis at different time points of 1 day, 3 days, and 5 days CM induction (0 h, 3 h, 6 h, 9 h). (Scale bar: 200 µm). **(B)** RT-qPCR analysis of VEGF mRNA expression changes in DPSCs after CM induction. **(C,D)** Number of crossing points and total tubule length in each group after 3 h of CM induction. **(E,F)** Number of crossover points and total tubule length in each group after 6 h of CM induction. (*P < 0.05; **P < 0.01; ***P < 0.001)

Dental pulp was a highly vascularized tissue, highlighting the value of angiogenesis for pulp regeneration (Zhang et al., 2024). *In vitro* tube-formation results (Figure 5A) indicated that DPSCs-secreted bioactive factors enabled the stimulation of angiogenesis. Endothelial cells in the control group showed a weak tendency to become vessels, and the addition of CM stimulation resulted in enhanced endothelial cell angiogenic capacity to different degrees. CM collected at different time points differed in their ability to promote endothelial cell vasculogenesis, with a weaker effect in the 1 day CM group, and a stronger ability of endothelial cells to form a complete tubular structure in the 3 days CM, and 5 days CM groups, revealing potent effect of vasculogenicity (*P* < 0.05). In regard of indicators of tubule-formation (junctions and tube network), the 3 days CM and 5 days CM groups were significantly higher than the control group (Figures 5C–F). Hence, the addition of active factors in CM promoted the vascularization of endothelial cells toward differentiation in a time-dependent manner. Moreover, the proangiogenic capacity of CM collected at 3 days and 5 days was superior to that of CM collected at 1 day. Furthermore, as presented in (Figure 5B), the mRNA expression levels of pro-angiogenesis-related genes in endothelial cells were significantly increased after CM treatment compared with the control. Hence, the addition of active factors in CM promoted the vascularization of endothelial cells toward differentiation in a time-dependent manner. Moreover, the proangiogenic capacity of CM collected at 3 days and 5 days was superior to that of CM collected at 1 day.

### 3.5 Subcutaneous implantation of DPSCs + CM + NPM injected tooth segments

**FIGURE 6:**
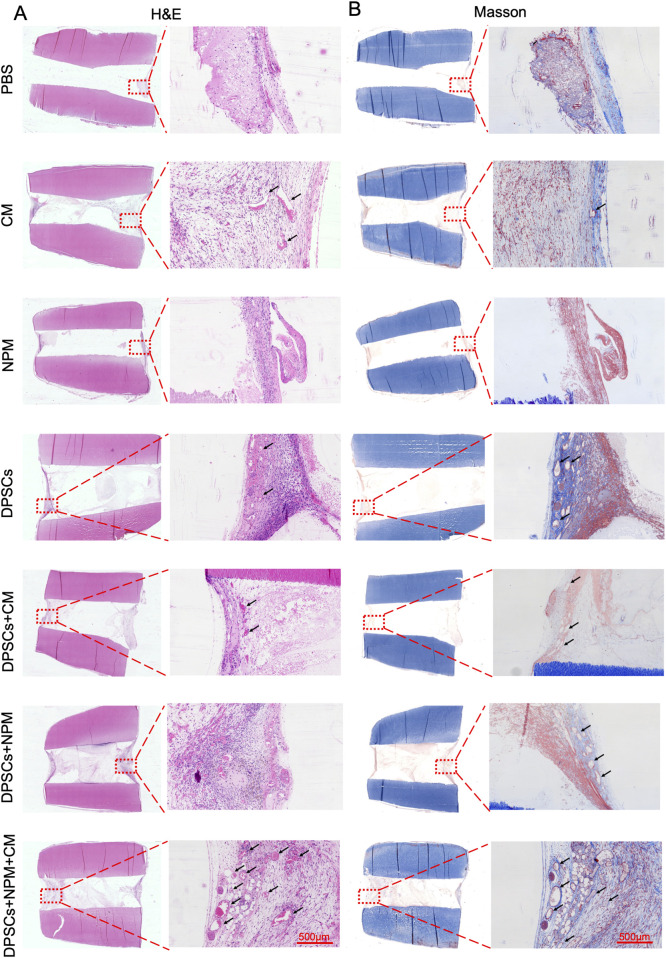
Dental pulp-like tissue regeneration by different materials combined with human root fragments after transplanting subcutaneously into immunodeficient mice for 8 weeks. **(A)** H&E images of paraffin sections from each group under light microscope. **(B)** Masson-stained images of paraffin sections from each group under light microscope. Typical areas were marked with red dotted frames in the leftmost images and the corresponding high magnification images were on the right (Scale bar: 500 µm).

To further simulate the tooth microenvironment, injections of PBS, CM, NPM, DPSCs, DPSCs+5dCM, DPSCs + NPM, DPSCs + NPM+5dCM were conducted into the root canal of the teeth and implanted into nude mice based on different grouping protocols, respectively. For the PBS, the NPM, and the CM groups with relatively little nascent tissue, the newly formed tissue was not tightly integrated with the inner dentin tubules (Figure 6). In contrast, the DPSCs, the DPSCs+5dCM, the DPSCs + NPM, and the DPSCs + NPM+5dCM groups showed a significantly higher amount of neotissue and blood vessels. Specifically, in the DPSCs + NPM+5dCM group, the regenerated pulp tissue was continuous, structurally intact, and tightly connected to dentin, with the observation of a large amount of neotissue and blood vessel formation, providing a better therapeutic effect compared with the other groups. In addition, similar results were found in both Masson and H&E staining. Moreover, Masson staining results revealed the formation of a well-structured pulp-like tissue in the DPSCs + NPM+5dCM group; and blue-stained collagen fiber formation in the neotissue. Therefore, the DPSCs + NPM+5dCM group had the best regeneration of pulp tissue.

## 4 Discussion

Building upon previous research, we have developed a new strategy for regenerating damaged dentin-pulp complex tissue using stem cell-based dental pulp regeneration (Li et al., 2024). Notably, Xuan et al. achieved successful pulp tissue regeneration featuring vascularization and sensory nerve integration by transplanting autologous stem cells from exfoliated deciduous teeth into young permanent teeth, which significantly advanced the research and clinical translation of this approach (Xuan et al., 2018). We propose that developing efficient cell delivery systems is crucial for expanding research in this field. In the present study, we pioneered the use of NPM as a scaffold material forDPSCs and CM delivery, demonstrating its feasibility for both *in vitro* and *in vivo* pulp regeneration applications.

Pulp tissue-derived stem cells are initially small in number, requiring post-extraction expansion in large quantities to meet therapeutic needs. Interestingly, scaffolding materials can be regarded as the “space station” for stem cell expansion *in vitro* and the “scaffolding” for the growth and function of walled stem cells (Wu et al., 2024). In endodontic regenerative tissue engineering, a major consideration would be the scaffold injectability (Guo et al., 2024). Root canals, featured by irregular and complex shape, challenge the filling of prefabricated scaffolds into the root canal system; moreover, multiple stresses and collisions may be triggered when injecting stem cells through a fine needle, finally inducing apoptosis (Qian et al., 2023). Significantly, the delivery of stem cells via microspheres can avoid direct cell injection-induced problems of low survival, cell damage, and cell death, being a reliable and protective engineering strategy (Khurshid et al., 2022). In the past, researches have successfully obtained allogeneic pulp decellularized scaffolds from human, porcine, or bovine dental pulp. Nevertheless, the fact is that it is still a great obstacle to collect abundant pulp decellularized scaffolds even when using large animal models. As mentioned previously, the basic problem is the limited size and number of pulp-decellularized scaffolds developed for clinical applications. Therefore, with the introduction of bovine pulp microspheres innovatively, 1% NPM were fabricated as injectable 3D macroporous bionic scaffolds for DPSCs delivery in our study. In view of their relatively uniform size and diameter of 350–450 μm, microspheres were suitable for oxygen diffusion and nutrient exchange, and could maintain the viability of DPSCs. An important structural feature of scaffolds is an interconnected porous structure, and the average pore size of the scaffolds should be large enough to promote cell migration. Here, 1% NPM showed lax and large pore structure, as shown by its fibrous porous structure, and relatively large pore size (35–60 μm), which was conducive to the adhesion of cells as well as their internal infiltration and growth. Therefore, 1% NPM was suitable for use as a cell delivery vehicle. In this study, DPSCs were grown into the porous wall of NPM, facilitating an enhanced proliferation of DPSCs and exhibiting cytoprotective potential during injection.

DPSCs have excellent neural differentiation and potent potential for angiogenesis, two characteristic factors for functional pulp regeneration, thus being the optimal source of cell for pulp regeneration. Activating factors can guide the directed pulp stem cell differentiation and promote blood vessel and nerve formation in the dental pulp, which is one of the key elements of pulp regeneration (Liang et al., 2024). Therefore, bioactive molecules are pivotal in the preparation of active factors possessing the ability of inducing pulp stem cell growth and differentiation, leading to functional pulp regeneration. Multiple types of active factors, exerting synergistic effects, exist in the regulation of pulp regeneration, and the use of a single growth factor has limited effects. As evidenced by existing studies, multiple growth factors can support pulp regeneration and that a single growth factor cannot fulfill the requirements for structural and functional pulp regeneration. Microspheres functionalized with platelet lysate (Zhang et al., 2021) or simvastatin (Yang et al., 2021) have been developed for pulp regeneration. However, multifunctional pulp tissue regeneration cannot be achievable as all microspheres with only a single growth factor functional modification, such as pro-angiogenic or pro-odontogenic differentiation property separately. It may highlight the feasibility of integrating different growth factors for pulp regeneration. As for the implementation of tissue engineering for pulp regeneration, the purpose lies in the development of a “cocktail” of growth factors to promote functional pulp tissue regeneration. CM for human DPSCs is rich in ontogenetically relevant bioactive factors, enabling angiogenesis, neurogenesis, and dental tissue repair (Sarra et al., 2021). Moreover, CM contains a series of secreted factors (e.g., growth factors, cytokines, extracellular vesicles, etc.) produced by DPSCs(Koh et al., 2022). On the basis of the functions of all these bioactive components, there may be impacts on cellular microenvironment as well as angiogenesis, immunomodulation, ECM remodeling and other processes related to tissue repair (de Cara et al., 2019). For instance, Zhou et al. (Zhou et al., 2021) demonstrated that CM from *ex vivo* cultured dental embryonic organs enhances DPSC proliferation, migration, *in vitro* mineralization, odontogenic differentiation, and angiogenesis, while also promotingearly *in vivo* pulp regeneration in animal models. Furthermore, preclinical studies have shown that DPSC-CM application enhances pulp regeneration and improves clinical outcomes.

Cell growth is one of the most critical factors in tissue regeneration. During the 7 days of cell culture in our study, the addition of CM from day 3 accelerated DPSC proliferation evidently, which peaked at day 7, and the promotion of DPSC proliferation was optimized by 5 days CM during culture. This indicated a positive effect of CM on cell proliferation. A key step in pulp regeneration is the differentiation of DPSCs into dentinogenic cells, which in turn form new dentin (Xie et al., 2024). Our study revealed higher adhesion and proliferation levels in DPSCs loaded on NPM. The porous structure of NPM and the enhanced cell adhesion resulted in higher viability of DPSCs after injection. In the odontogenically induced microenvironment, CM promoted DPSCs to differentiate odontogenically. CM-stimulated ALP activity of DPSCs was highly elevated and induced more calcium nodule formation. In addition, CM promoted the expression of angiogenic genes in DPSCs, which effectively contributed to the tubule formation and neovascular regeneration of HUVECs. Subsequently, our study determined the biological properties of medullary microsphere scaffolds, and identified multiple differentiation functions of the transplanted pulp stem cells through implantation of DPSCs + NPM + CM complexes in nude mice. *In vivo* experiments, considering the low cell retention and survival, less regenerative tissue formation was found in the DPSCs suspension group. In contrast, microsphere-loaded DPSCs effectively increased the potential for complex integration with host tissues, demonstrating the effect of stem cell administration in enhancing scaffold integration into host tissues *in vivo*. After injecting DPSCs + NPM + CM complexes, more vascularized tissues with pulp cell-like phenotype were successfully formed in nude mice. Consequently, our study, to this extent, demonstrated the practicability of the NPM stem cell-loaded and CM-released system for vascularized pulp regeneration.

This study has several limitations that need to be addressed. First, although dental pulp stem cell-conditioned medium (CM) contains various growth factors, fluctuations in the concentration of specific bioactive components may affect the stability of regenerative outcomes. This issue requires further clarification through proteomic analysis. Second, the absence of newly formed dentin observed in H&E-stained sections may be attributed to an insufficient number of transplanted stem cells (Li et al., 2016; Silva et al., 2018),a limitation that warrants improvement in future studies. Additionally, more advanced techniques are needed to systematically evaluate the formation and function of new blood vessels and to track the fate of transplanted DPSCs, such as microangiography (Atlas et al., 2021) and cell tracing technologies (Biz et al., 2020). The combination of cryopreservation technology with cell-laden microspheres presents a promising method for the storage of cells and micro-tissues, which can facilitate clinical translation and commercialization in the field of dental pulp regeneration. Further research on the cryopreservation of cell-loaded pulp microspheres will be required in the future (Chang et al., 2017). For further clinical translation, the DPSCs + NPM + CM complexes should be injected into full length root canals sealed with optimal seal materials to explore the regenerative capacity *in vivo*. Finally, future studies should prioritize establishing large animal models for *in situ* dental pulp regeneration experiments.

## 5 Conclusion

In this work, we found that CM can promote dentinogenic differentiation and angiogenesis of DPSCs. NPM was introduced as a novel alternative scaffold to effectively deliver DPSCs and CM into irregularly shaped narrow root canals, and it facilitates DPSCs adhesion and cytoprotection during injection. The DPSCs + NPM + CM complexes showed enhanced odontogenic and proangiogenic potential *in vitro*. Furthermore, the DPSCs + NPM + CM complexes enhanced microvessel formation and pulp-like tissue regeneration *in vivo*. Collectively, this study demonstrates the broad application potential of NPM as a tissue engineering scaffold material for dental pulp regeneration.

## Data Availability

The datasets presented in this study can be found in online repositories. The names of the repository/repositories and accession number(s) can be found in the article/Supplementary Material.
