# Supplementary material for: Dental pulp stem cells conditioned medium-functionalized microspheres for endodontic regeneration
Source: Front Cell Dev Biol. 2025 Jul 17;13:1627220. doi: 10.3389/fcell.2025.1627220 (PMC12310587; doi:10.3389/fcell.2025.1627220)
Supplement: Supplementary file 1 [file DataSheet1.docx]

1. **Supplementary Table**

Table S1. DNA sequences used in this work

| Target cDNA | Primer sequence (5′−3′) |
| --- | --- |
| GAPDH-Forward | GCACCGTCAAGGCTGAGAAC |
| GAPDH-Reverse | TGGTGAAGACGCCAGTGGA |
| DSSP-Forward | TGGCGATGCAGGTCACAAT |
| DSSP-Reverse | CCATTCCCACTAGGACTCCCA |
| DMP-1-Forward | CACTCAAGATTCAGGTGGCAG |
| DMP-1-Reverse | TCTGAGATGCGAGACTTCCTAAA |
| VEGF-Forward | AGGGCAGAATCATCACGAAGT |
| VEGF-Reverse | AGGGTCTCGATTGGATGGCA |

1. **Supplementary Figures**


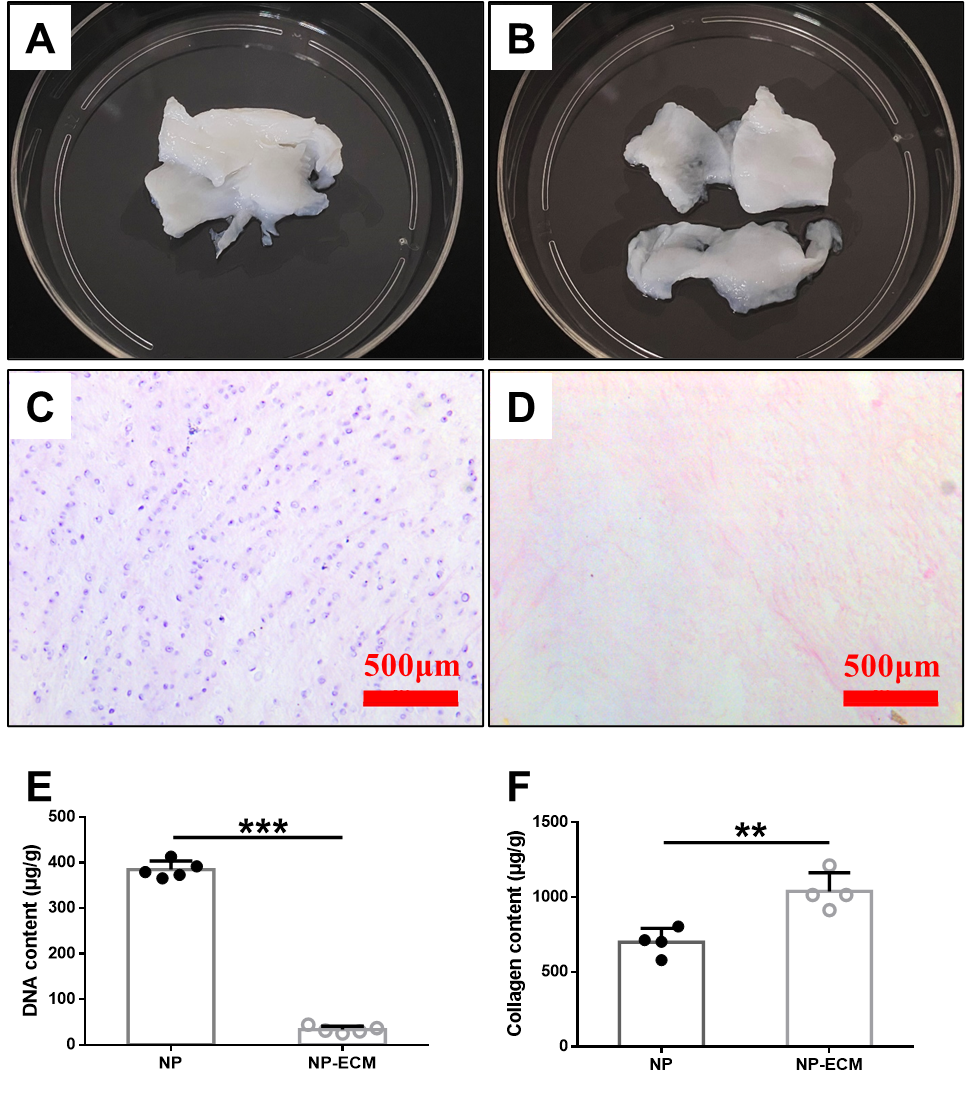


Figure S1. Characterization of decellularized nucleus pulposus tissue. (A, B) Gross appearance of nucleus pulposus tissue before and after decellularization. (C, D) H&E staining images of nucleus pulposus tissue before and after decellularization. (E) Statistical results of DNA quantitative analysis. (F) Quantitative analysis results of collagen. NP native nucleus pulposus, NP-ECM decellulaized nucleus pulposus-ECM, *** *P*＜0.001, ** *P*＜0.01.


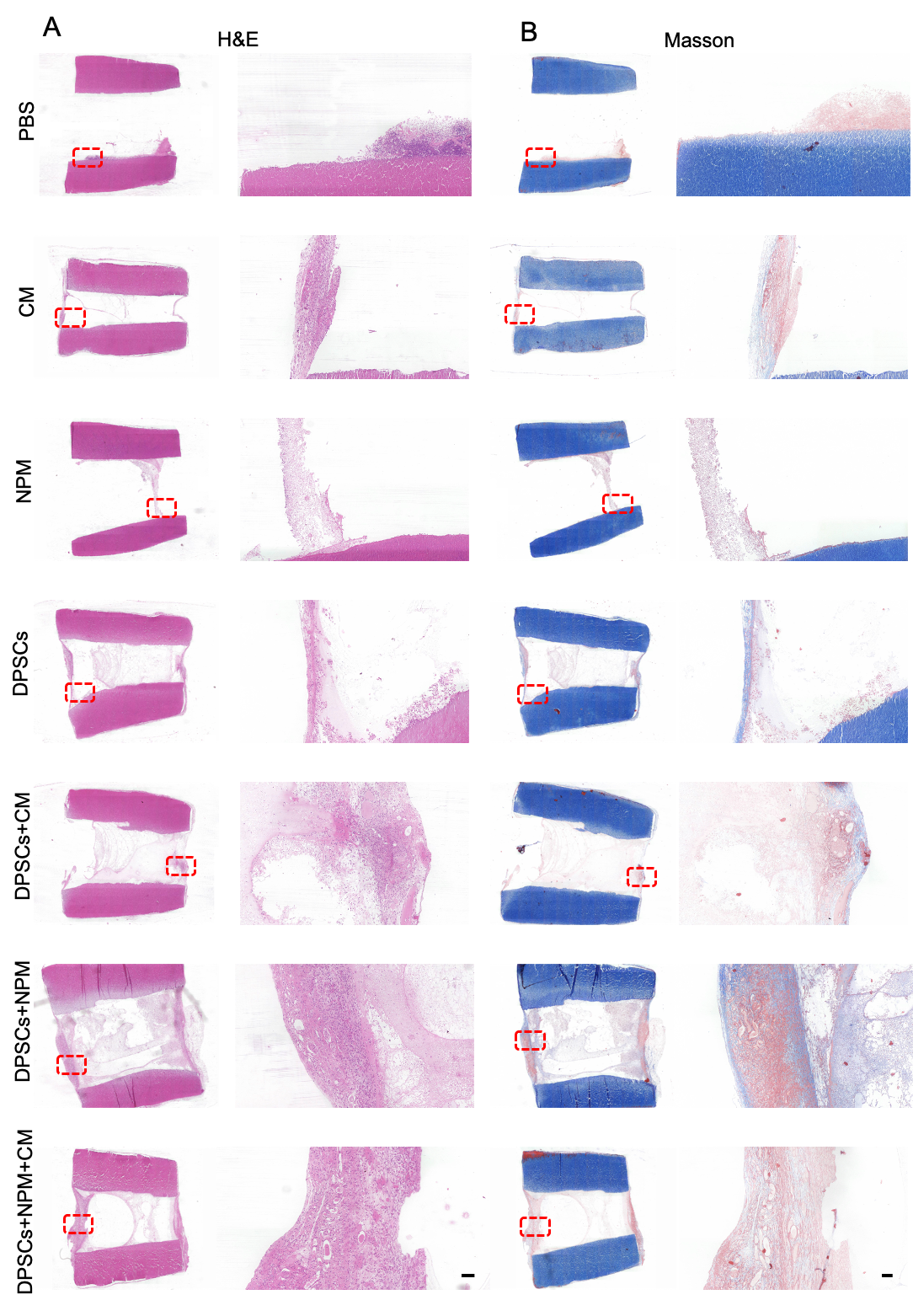


Figure S2. Dental pulp-like tissue regeneration by different materials combined with human root fragments after transplanting subcutaneously into immunodeficient mice for 4 weeks. (A) H&E images of paraffin sections from each group under light microscope. (B) Masson-stained images of paraffin sections from each group under light microscope. Typical areas were marked with red dotted frames in the leftmost images and the corresponding high magnification images were on the right(Scale bar: 100µm).
